# Cigarette smoke and lipopolysaccharide induce a proliferative airway smooth muscle phenotype

**DOI:** 10.1186/1465-9921-11-48

**Published:** 2010-04-29

**Authors:** Tonio Pera, Reinoud Gosens, Andries H Lesterhuis, Riham Sami, Marco van der Toorn, Johan Zaagsma, Herman Meurs

**Affiliations:** 1 Department of Molecular Pharmacology, University Centre for Pharmacy, University of Groningen, Groningen, The Netherlands; 2 Laboratory of Allergology and Pulmonary Diseases, University Medical Center Groningen, Groningen, The Netherlands

## Abstract

**Background:**

A major feature of chronic obstructive pulmonary disease (COPD) is airway remodelling, which includes an increased airway smooth muscle (ASM) mass. The mechanisms underlying ASM remodelling in COPD are currently unknown. We hypothesized that cigarette smoke (CS) and/or lipopolysaccharide (LPS), a major constituent of CS, organic dust and gram-negative bacteria, that may be involved in recurrent airway infections and exacerbations in COPD patients, would induce phenotype changes of ASM.

**Methods:**

To this aim, using cultured bovine tracheal smooth muscle (BTSM) cells and tissue, we investigated the direct effects of CS extract (CSE) and LPS on ASM proliferation and contractility.

**Results:**

Both CSE and LPS induced a profound and concentration-dependent increase in DNA synthesis in BTSM cells. CSE and LPS also induced a significant increase in BTSM cell number, which was associated with increased cyclin D1 expression and dependent on activation of ERK 1/2 and p38 MAP kinase. Consistent with a shift to a more proliferative phenotype, prolonged treatment of BTSM strips with CSE or LPS significantly decreased maximal methacholine- and KCl-induced contraction.

**Conclusions:**

Direct exposure of ASM to CSE or LPS causes the induction of a proliferative, hypocontractile ASM phenotype, which may be involved in airway remodelling in COPD.

## Background

Chronic obstructive pulmonary disease (COPD) is an inflammatory lung disease characterized by a progressive and largely irreversible airflow obstruction, which involves structural changes of the lung, including emphysema and small airway remodelling [1]. Small airway remodelling in COPD is characterized by adventitial fibrosis and mucus cell hyperplasia, and may involve increased airway smooth muscle (ASM) mass, particularly in severe disease [1-5]. Small airway remodelling may contribute to the reduced lung function as well as to persistent airway hyperresponsiveness, which is present in most of the patients [6,7].

Tobacco smoke exposure is considered to be the most important risk factor for COPD in developed countries. Lipopolysaccharide (LPS) - a constituent of the outer wall of gram-negative bacteria and a contaminant of tobacco smoke, organic dust and environmental pollution [8-11] - has been implicated in the development and progression of various pulmonary diseases, including COPD [12-14]. Cigarette smoke (CS) and LPS have previously been shown to induce features of airway remodelling in animal models, including airway wall thickening, increased ASM mass, goblet cell hyperplasia and collagen deposition [15-19].

Although the mechanisms involved in the development and progression of small airway remodelling in COPD are largely unknown, chronic inflammation of the airways is presumably of major importance. This is indicated by persistent infiltration of inflammatory cells, including macrophages, neutrophils and T- and B-lymphocytes, in the airway wall, which is correlated with the severity of airflow obstruction [3,5].

This inflammatory response is associated with the release of profibrotic cytokines and growth factors, which are linked to a repair and remodelling process that thickens the airway wall and narrows the airway lumen [20].

However, small airway remodelling could also result from direct effects of CS and LPS exposure on structural cells of the airway wall, independent of inflammation. Thus, studies using rat tracheal explants [21,22] and a mouse model of CS exposure [23] have shown that CS exposure of the airway wall may lead to the release of TGF-β_1 _and upregulation of platelet-derived growth factor (PDGF), connective tissue growth factor (CTGF) and procollagen gene expression independent of inflammatory cell infiltration. The inflammation-independent fibrotic response presumably involves an oxidant-driven mechanism, which may be reinforced by inflammatory cells such as macrophages and neutrophils, known to release oxidants in response to tobacco smoke [24]. In addition, epithelial cells, fibroblasts, as well as ASM cells in culture have been shown to release pro-inflammatory and profibrotic cytokines in response to CS [25-29] or LPS [30-32].

As indicated above, various studies have indicated that increased airway smooth muscle mass may contribute to airway remodelling in COPD [2-5]. Indeed, a direct correlation between the degree of smooth muscle mass and airflow obstruction in COPD has been reported [3,5]. Previous in vitro studies from our laboratory have demonstrated that growth factors, including PDGF, and extracellular matrix (ECM) proteins, including collagen I and fibronectin, induce a proliferative phenotype of bovine tracheal smooth muscle (BTSM), which is accompanied by reduced contractility of the muscle [33-35]. PDGF-induced phenotypic modulation was shown to be mediated by ERK 1/2 and p38 MAP kinase, two signalling molecules that are importantly involved in mitogenic responses of ASM [33,35]. The direct effects of CSE and LPS on ASM proliferation are, however, currently unknown. In this study, we present evidence that both CSE and LPS induce a proliferative, hypocontractile phenotype of ASM independent of inflammation, which could be important in the development and progression of ASM growth in COPD.

## Methods

### Isolation of Bovine Tracheal Smooth Muscle Cells

Bovine tracheae were obtained from local slaughterhouses and transported to the laboratory in Krebs-Henseleit buffer of the following composition (mM): NaCl 117.5, KCl 5.60, MgSO_4 _1.18, CaCl_2 _2.50, NaH_2_PO_4 _1.28, NaHCO_3 _25.00, and glucose 5.50, pregassed with 5% CO_2 _and 95% O_2_; pH 7.4. After dissection of the smooth muscle layer and removal of mucosa and connective tissue, tracheal smooth muscle was chopped using a McIlwain tissue chopper, three times at a setting of 500 μm and three times at a setting of 100 μm. Tissue particles were washed two times with Dulbecco's Modified Eagle's Medium (DMEM), supplemented with NaHCO_3 _(7 mM), HEPES (10 mM), sodium pyruvate (1 mM), nonessential amino acid mixture (1:100), gentamicin (45 μg/ml), penicillin (100 U/ml), streptomycin (100 μg/ml), amphotericin B (1.5 μg/ml), and foetal bovine serum (FBS, 0.5%) (all purchased from GIBCO BRL Life Technologies, Paisley, UK). Enzymatic digestion was performed using the same medium, supplemented with collagenase P (0.75 mg/ml, Boehringer, Mannheim, Germany), papain (1 mg/ml, Boehringer), and Soybean trypsin inhibitor (1 mg/ml, Sigma Chemical, St. Louis, MO, USA). During digestion, the suspension was incubated in an incubator shaker (Innova 4000) at 37°C, 55 rpm for 20 min, followed by a 10-min period of shaking at 70 rpm. After filtration of the obtained suspension over a 50 μm gauze, cells were washed three times in supplemented DMEM containing 10% FBS. This isolation method results in a cell population positive for smooth muscle α-actin (95%) and smooth muscle myosin heavy chain [33,36].

### Cigarette Smoke Extract

Cigarette smoke extract was prepared by combusting 2 research cigarettes (University of Kentucky 2R4F; filters removed), using a peristaltic pump (Watson Marlow 323 E/D, Rotterdam, The Netherlands) and passing the smoke through 25 ml of FBS-free DMEM supplemented with penicillin and streptomycin at a rate of 5 minutes/cigarette. The obtained solution is referred to as 100% strength.

### [^3^H]-Thymidine Incorporation

BTSM cells were plated in 24-well cluster plates at a density of 50,000 cells per well, and were allowed to attach overnight in 10% FBS-containing DMEM at 37°C in a humidified 5% CO_2 _incubator. Cells were washed two times with sterile phosphate-buffered saline (PBS, composition [mM] NaCl, 140.0; KCl, 2.6; KH_2_PO_4_, 1.4; Na_2_HPO_4_.2H_2_O, 8.1; pH 7.4) and made quiescent by incubation in FBS-free medium, supplemented with apo-transferrin (5 μg/ml, human, Sigma), ascorbate (100 μM, Merck, Darmstadt, Germany), and insulin (1 μM, bovine pancreas, Sigma) for 72 h. Cells were then washed with PBS and stimulated with LPS (1-10,000 ng/ml), purified from *Escherichia coli *O55:B5 (Sigma) or PDGF (10 ng/ml) in FBS-free medium for 28 h. Treatment of cells with CSE (1-50%) lasted 1 h, after which the cells were washed 3 times with PBS and incubated in FBS-free DMEM for another 27 h. [^3^H]-thymidine (0.25 μCi/ml, Amersham, Buckinghamshire, UK) was present during the last 24 h of the incubations, followed by two washes with PBS at room temperature and one wash with ice-cold 5% trichloroacetic acid (TCA). Cells were incubated with TCA on ice for 30 min. Subsequently, the acid-insoluble fraction was dissolved in 0.5 ml NaOH (1 M). Incorporated [^3^H]-thymidine was quantified by liquid-scintillation counting.

### Cell number determination

BTSM cells were plated in 6-well cluster plates at a density of 100,000 cells/well in medium, containing 10% FBS. Cells were grown to 50% confluence after which they were serum-deprived for 72 h. Subsequently, cells were treated with CSE (15%) 2 times for 1 h, on day 0 and day 2, respectively, or with LPS (1 μg/ml) or PDGF (10 ng/ml) for 4 days continuously. On day 4, the cells were washed twice with PBS and were trypsinized (0.25% Trypsin-EDTA (GIBCO); 15 min) and re-suspended in FBS-containing DMEM. Cells were then counted in duplicate, using a hemocytometer. When applied, the MEK inhibitors U0126 (3 μM; Tocris Cookson, Bristol, UK) or PD 98059 (30 μM, Sigma) and the p38 MAPK inhibitors SB 203580 (10 μM, Tocris) or SB 239063 (10 μM, Sigma) were added to the cells 30 min before stimulation and were present throughout the experiment.

### Western blot analysis

BTSM cells were plated in 6-well cluster plates at a density of 200,000 cells/well in medium, containing 10% fetal bovine serum. Upon confluence, cells were washed two times with sterile PBS and made quiescent by incubation in serum-free medium, supplemented with apo-transferrin (5 μg/ml) and ascorbate (100 μM) for either 24 h, for ERK 1/2 and p38 MAP kinase phopsphorylation, or 72 h, for cyclin D1 expression. Cells were then washed with PBS and stimulated in serum-free medium. To obtain total cell lysates, cells were washed once with ice-cold phosphate-buffered saline (PBS) and then lysed in ice-cold RIPA buffer (composition: 50 mM Tris, 150 mM NaCl, 1% Igepal CA-630, 1% deoxycholic acid, 1 mM NaF, 1 mM Na_3_VO_4_, 10 μg/ml aprotinin, 10 μg/ml leupeptin, 7 μg/ml pepstatin A, 5 mM 2-glycerophosphoric acid, pH 8.0). Lysates were stored at -80°C until further use. Cultured tissue strip homogenates were prepared by pulverizing the tissue under liquid nitrogen, followed by sonification in ice-cold RIPA buffer. Protein content was determined according to Bradford [37]. Homogenates containing 50 μg of protein per lane were then subjected to immunoblot analysis using antibodies against cyclin D1, ERK 1/2, p38 MAP kinase or the phosphorylated forms of ERK 1/2 (Thr^202^/Tyr^204^) or p38 MAP kinase (Thr^180^/Tyr^182^) (Cell Signaling Technology, Beverly, MA, USA). The antibodies were visualized using enhanced chemiluminescence. Photographs of the blots were scanned and analyzed by densitometry (Totallab™; Nonlinear Dynamics, Newcastle, UK).

### Tissue culture

After dissection of the smooth muscle layer and careful removal of mucosa and connective tissue, tracheal smooth muscle strips were prepared while incubated in gassed KH-buffer at room temperature. Care was taken to cut tissue strips with macroscopically identical length (1 cm) and width (2 mm). Tissue strips were washed once in sterile FBS-free DMEM, supplemented with apo-transferrin (5 μg/ml) and ascorbate (100 μM). Next, the tissue strips were transferred into suspension culture flasks containing a volume of 7.5 ml medium. CSE treated strips were exposed to 15% CSE for 1 h daily during 8 days. LPS treatment was performed in the continuous presence of 1 μg/ml LPS during 8 days.

### Isometric tension measurements

Tissue strips, collected from the suspension culture flasks, were washed with several volumes of KH buffer pregassed with 5% CO_2 _and 95% O_2_, pH 7.4 at 37°C. Subsequently, the strips were mounted for isometric recording (Grass force-displacement transducer FT03) in 20-ml water-jacked organ baths containing KH buffer at 37°C, continuously gassed with 5% CO_2 _and 95% O_2_, pH 7.4. During a 90-min equilibration period, with washouts every 30 min, resting tension was gradually adjusted to 3 g. Subsequently, the muscle strips were precontracted with 20 and 40 mM isotonic KCl solutions. Following two washouts, maximal relaxation was established by the addition of 0.1 μM (-)-isoprenaline (Sigma). In most of the experiments, no basal myogenic tone was detected. Tension was readjusted to 3 g, immediately followed by three washes with fresh KH buffer. After another equilibration period of 30 min, cumulative concentration response curves were constructed using stepwise increasing concentrations of isotonic KCl (5.6-50 mM) or methacholine (1 nM-100 μM; ICN Biomedicals, Costa Mesa, CA, USA). When maximal tension was obtained, the strips were washed several times, and maximal relaxation was established using 10 μM (-)-isoprenaline.

### Data analysis

All data represent means ± s.e. mean from separate experiments. The statistical significance of differences between data was determined by the Student's t-test for paired observations. Differences were considered to be statistically significant when P < 0.05.

## Results

### CSE and LPS induce BTSM cell proliferation

**Figure 1:**
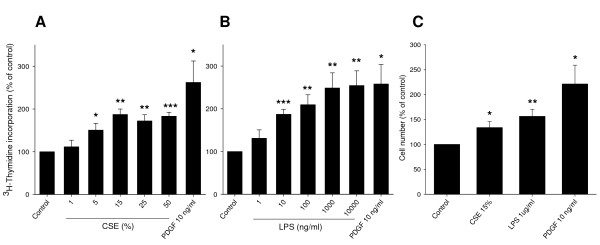
**CSE and LPS induce BTSM cell proliferation**. Subconfluent, serum-deprived BTSM cells were treated with increasing concentrations of CSE for 1 h (A) or increasing concentrations of LPS for 28 h (B). [^3^H]-Thymidine incorporation was determined 28 h after stimulation as described under methods. Data represent means ± S.E.M. of 5-7 experiments, each performed in triplicate. (C) Serum-deprived BTSM cells were treated 2 times (1 h, day 0 and day 2) with CSE (15%) or 4 days with LPS (1 μg/ml) or PDGF (10 ng/ml). Cells were counted in duplicate on day 4, using a hemocytometer. Data represent means ± S.E.M. of 5-8 experiments. *P < 0.05, **P < 0.01, ***P < 0.001 vs control

Proliferative responses of isolated BTSM cells to CSE and LPS stimulation were investigated by [^3^H]-thymidine incorporation and cell counting. A 1 h pulse treatment with CSE, followed by 27 h incubation in serum-free medium resulted in a significant and concentration-dependent increase in [^3^H]-thymidine incorporation, reaching a maximum of 187 ± 13% of control at a concentration of 15% (Figure 1A). Similarly, LPS induced a concentration-dependent increase in [^3^H]-thymidine incorporation of up to 254 ± 45% of control, similar to that induced by a submaximal concentration of PDGF (10 ng/ml; 258 ± 64%) (Figure 1B). Treatment of BTSM cells with 15% CSE (two 1 h pulses, on day 0 and day 2), or 1 μg/ml LPS resulted in a significant increase in cell number as well, as determined 4 days after starting the treatment (Figure 1C). As a positive control, PDGF (10 ng/ml, 4 days) similarly increased BTSM cell number (Figure 1C). The combined treatment of cells with CSE (15%) and LPS (1 μg/ml) had no additional effect on cell numbers when compared to the separate treatments alone (data not shown). Collectively, these data indicate that both CSE and LPS induce proliferation of BTSM cells in a non-additive fashion.

### CSE and LPS induce ERK 1/2 and p38 MAP kinase phosphorylation and cyclin D1 expression

**Figure 2:**
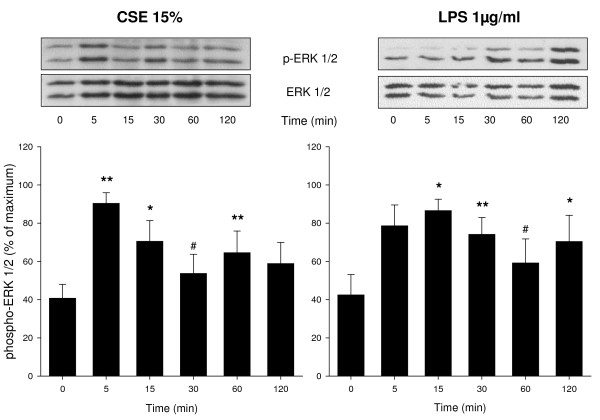
**CSE and LPS induce ERK 1/2 phosphorylation**. Serum deprived BTSM cells were treated with CSE (15%) or LPS (1 μg/ml) up to 2 h. Cell lysates were analyzed by immunoblotting for phospho-ERK 1/2 (Thr^202^/Tyr^204^) and total ERK 1/2 to correct for differences in protein loading. Phospho-ERK 1/2 was quantified using densitometry and normalized to the maximal response in each experiment. Representative blots are shown. Data represent means ± S.E.M. of 7 experiments. *P < 0.05, **P < 0.01, ^#^P < 0.1 vs control at t = 0

**Figure 3:**
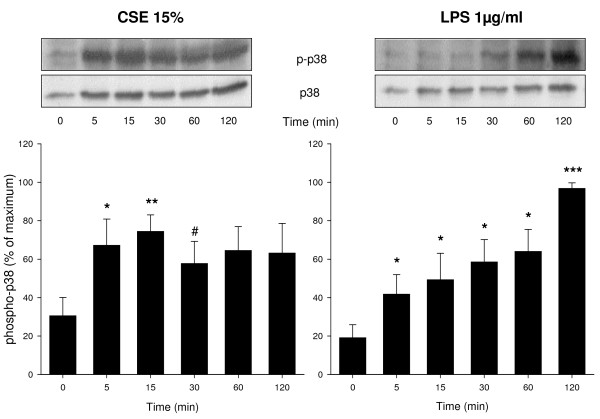
**CSE and LPS induce p38 MAP kinase phosphorylation**. Serum deprived BTSM cells were treated with CSE (15%) or LPS (1 μg/ml) up to 2 h. Cell lysates were obtained and analyzed by immunoblotting for phospho-p38 MAP kinase (Thr^180^/Tyr^182^) and total p38 MAP kinase to correct for differences in protein loading. Immunoblots were quantified using densitometry and the abundance of CSE- or LPS-induced p38 MAP kinase phosphorylation was normalized to the maximal response in each individual experiment. Representative blots are shown. Data represent means ± S.E.M. of 4-5 experiments. *P < 0.05, **P < 0.01, ***P < 0.001, ^#^P < 0.1 vs control at t = 0

**Figure 4:**
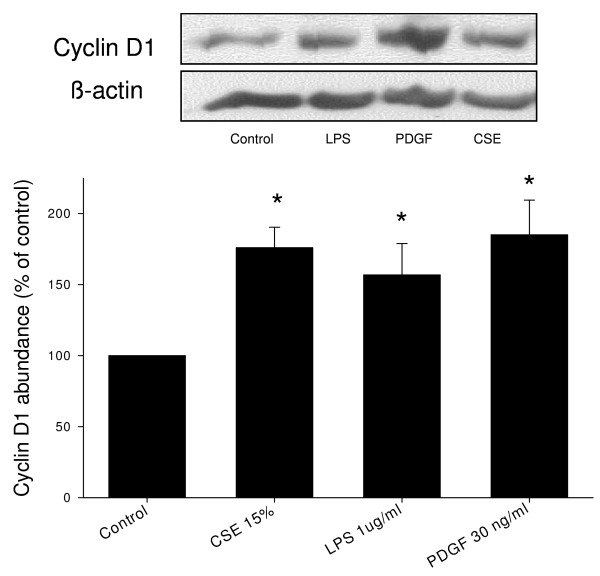
**CSE and LPS increase cyclin D1 expression**. Serum-deprived BTSM cells were treated with CSE (15%) for 1 h, or LPS (1 μg/ml) or PDGF (30 ng/ml) for 24 h. Cell lysates were obtained 24 h after stimulation and analyzed by immunoblotting for cyclin D1 and β-actin to correct for protein loading. Cyclin D1 was quantified using densitometry and normalized to control expression. Data represent means ± S.E.M. of 4-7 experiments. *P < 0.05 vs control

Western blot analysis was performed to investigate the effects of CSE (15%) and LPS (1 μg/ml) on phosphorylation of ERK 1/2 and p38 MAP kinase, two major signalling pathways involved in ASM cell proliferation, and on the expression of cyclin D1, a key regulator of cell cycle progression downstream of ERK 1/2 and p38 MAP kinase. Both CSE and LPS induced a rapid phosphorylation of ERK 1/2 (Figure 2). Both stimuli also induced a rapid phosphorylation of p38 MAP kinase, which, similarly to ERK 1/2 phosphorylation, was sustained (Figure 3). In addition, both CSE and LPS significantly increased the expression of cyclin D1, as assessed after 24 h, to a similar extent as 30 ng/ml PDGF (Figure 4), suggesting an important role for these signalling pathways in the proliferative response induced by CSE and LPS.

### Role of ERK 1/2 and p38 MAP kinase in CSE- and LPS-induced proliferation

**Figure 5:**
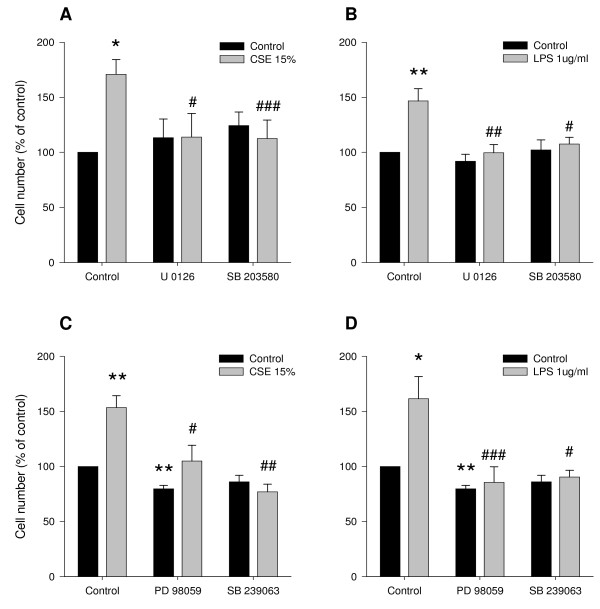
**CSE- and LPS-induced proliferation is dependent on ERK 1/2 and p38 MAP kinase**. Serum-deprived BTSM cells were treated 2 times (1 h, day 0 and day 2) with CSE (15%) or 4 days continuously with LPS (1 μg/ml) in the absence or presence of the MEK inhibitor U0126 (3 μM) and the p38 MAP kinase inhibitor SB 203580 (10 μM) (panels A and B), as well as in the absence or presence of the MEK inhibitor PD 98059 (30 μM) and the p38 MAP kinase inhibitor SB 239063 (10 μM) (panels C and D). Cells were counted in duplicate on day 4, using a hemocytometer. Data represent means ± S.E.M. of 4-7 experiments. *P < 0.05, **P < 0.01 vs untreated control, ^#^P < 0.05, ^##^P < 0.01, ^###^P < 0.001 vs CSE or LPS treatment in the absence of inhibitor.

To test this hypothesis, the effect of CSE or LPS on cell number was determined in the presence or absence of U0126 (3 μM), an inhibitor of MEK, the upstream activator of ERK 1/2, or SB 203580 (10 μM), an inhibitor of p38 MAP kinase. As illustrated in Figures 5A and 5B, inhibition of MEK by U0126 and inhibition of p38 MAP kinase by SB 203580 completely abrogated the CSE- and LPS-induced increase in cell number. By contrast, no effect of the kinase inhibitors on basal cell numbers was observed. These findings were confirmed by using PD 98059 (30 μM) and SB 239063 (10 μM), alternative inhibitors for MEK and p38 MAP kinase, respectively (Figures 5C and 5D). Together with the CSE- and LPS-induced phosphorylation of ERK 1/2 and p38 MAP kinase described above, these data indicate that CSE- and LPS-induced proliferation is dependent on activation of the ERK 1/2 and p38 MAP kinase signalling pathways.

### Effects of LPS and CSE on BTSM contractility

**Figure 6:**
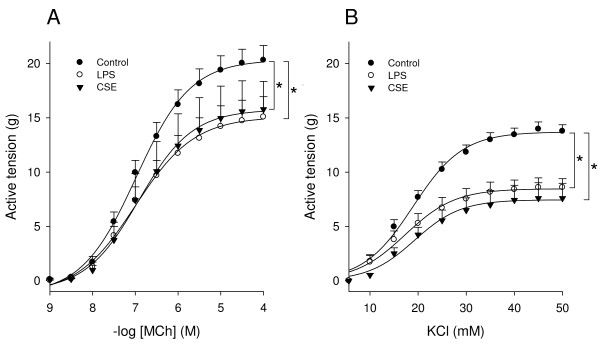
**CSE and LPS induce BTSM hypocontractility**. Methacholine (A)- and KCl (B)-induced contractions of BTSM strips cultured for 8 days with or without LPS (1 μg/ml) or exposed to 15% CSE for 1 h daily during 8 days. Data represent means ± S.E.M. of 4-6 experiments, each performed in duplicate. *P < 0.05 vs control.

**Figure 7:**
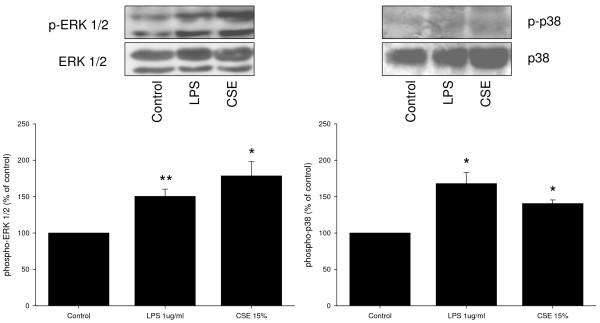
**CSE and LPS induce ERK 1/2 and p38 MAP kinase phosphorylation in BTSM strips**. BTSM strips were cultured for 8 days with or without LPS (1 μg/ml) or exposed to 15% CSE for 1 h daily during 8 days. Tissue lysates were analyzed by immunoblotting for phospho-ERK 1/2 (Thr^202^/Tyr^204^) and phospho-p38 MAP kinase (Thr^180^/Tyr^182^). Differences in protein loading were corrected for by immunoblotting for total ERK 1/2 and total p38 MAP kinase. Immunoblots were quantified using densitometry. The abundance of CSE- and LPS-induced ERK 1/2 and p38 MAP kinase phosphorylation was normalized to controls from untreated strips. Data represent means ± S.E.M. of 3-6 experiments. *P < 0.05, **P < 0.01 vs control

Previous studies have shown that the proliferative response of BTSM cells to growth factors and ECM proteins is linearly related to a decrease in contractility of BTSM tissue [33,34]. In order to investigate the effects of CSE and LPS on BTSM phenotype, strips were cultured for 8 days with 1 μg/ml LPS or were subjected to daily exposure to 15% CSE for 1 h during 8 days. After both treatments, maximal contraction induced by methacholine or KCl was significantly reduced compared to untreated strips (Figures 6A and 6B). No differences in the sensitivity (-log EC_50_) to methacholine and KCl were found. These effects were associated with increased ERK 1/2 and p38 MAP kinase phosphorylation in the tissue (Figure 7). Collectively, these results indicate that both CSE and LPS induce a shift to a hypocontractile and proliferative ASM phenotype.

## Discussion

In this study, we demonstrated for the first time that CSE and LPS induce a profound and concentration-dependent increase in DNA synthesis and cell number of cultured ASM cells. The CSE- and LPS-induced proliferation is dependent on phosphorylation of ERK 1/2 and p38 MAP kinase and downstream mitogenic signalling. In addition, we demonstrated that CSE and LPS treatments reduce the maximal contraction of ASM preparations to methacholine and KCl, which is also associated with increased ERK 1/2 and p38 MAP kinase phosphorylation. Collectively, these data indicate that CSE and LPS induce a phenotype shift of ASM to a proliferative and less contractile phenotype that could be involved in airway remodelling in COPD.

Although small airway remodelling has been associated with cellular inflammation, evidence suggesting that direct action of cigarette smoke on the airway wall is involved in airway remodelling is accumulating. In rat tracheal explants, Wang and colleagues [21,22] demonstrated direct effects of CS on the release of active TGF-β_1_, with subsequent phosphorylation of Smad-2 and upregulation of CTGF and procollagen gene expression. In addition, in a cell-free system, cigarette smoke extract was found to release active TGF-β_1 _from (recombinant) latent TGF-β_1 _via an oxidative mechanism [22]. Acute CS exposure of mice may also induce a transient increase in TGF-β_1_-, CTGF-, procollagen- and PDGF-gene expression and Smad-2 phosphorylation [23]. While the maximal response was observed 2 h after CS exposure, the increase in inflammatory cell numbers was only significant after 24 h, by which time the gene expression had subsided. This indicates that a dissociation between pro-fibrotic remodelling responses and inflammatory cell responses may occur. Chronic CS exposure of these mice resulted in a persistent increase in gene expression of above-mentioned factors and an increase in airway wall collagen. Collectively, these data indicate that CS may initiate airway remodelling by inducing profibrotic growth factors in the airway wall, which can lead to increased deposition of matrix proteins. In addition, these observations imply that CS creates conditions which are strongly mitogenic to ASM, since both growth factors and collagen promote ASM proliferation, which may lead to an increase in ASM mass [33,34,38]. Our present observations indicate that a direct effect of CS on ASM proliferation may also be involved in airway remodelling. To what extent autocrine processes, involving the release of growth factors and/or pro-proliferative ECM proteins by these cells [39,40], may play a role, is currently unknown. Remarkably, previous reports [41] have indicated that CSE may also augment proliferation of passively sensitized human ASM cells.

Prolonged exposure of cultured airway structural cells, including ASM cells, to CSE may have cytotoxic effects on these cells by inducing apoptosis and necrosis in a concentration- and time-dependent manner [42-45]. Thus, in human ASM cells, a time- and concentration-dependent induction of cell-cycle arrest, apoptosis and necrosis by exposure to 2,5 - 20% CSE for 24 - 72 h has been demonstrated [42]. Accordingly, the viability of our BTSM cells was reduced after 24 h continuous incubation of the cells with 15% CSE (not shown). However, it was found that short, pulsed exposures of ASM cells to 5 - 50% CSE have a proliferative rather than a toxic effect on these cells. This is of major importance, as this approach seems to be a more suitable model for mimicking the *in vivo *effects of CS than continuous exposure to high concentrations of CSE for several hours. In addition, CSE exposure may be a more suitable approach for studying the direct, epithelium-independent effects of CS on ASM, as during smoking ASM is not directly exposed to CS but indirectly, to components of CS after passing the epithelial barrier.

LPS activates the Toll-like receptor 4 (TLR4) signalling pathway, causing activation NF-κB and AP1, which results in transcription of pro-inflammatory cytokine genes and initiation of the innate immune response [46]. In human subjects, acute experimental LPS inhalation leads to pulmonary and systemic inflammatory responses associated with airways obstruction and increased airway responsiveness [47,48]. Chronic exposure to LPS-containing dust or bio-aerosol in occupational or home environment has also been associated with persistent airway inflammation, decline of lung function and airway hyperresponsiveness [14,49,50]. Moreover, LPS exposure may contribute to the severity of asthma [50]. LPS may be importantly involved in bacterial infection-induced exacerbations of COPD, which contribute to the progression of the disease and diminish the quality of life [51-53]. In animal models, exposure to LPS induces various inflammatory and pathological changes closely mimicking COPD, including airway remodelling and emphysema [17,18,54]. Our present data provide evidence that a direct effect of LPS on ASM cell proliferation may contribute to airway remodelling. Although it has been reported that tobacco smoke is contaminated with LPS [8], LPS is unlikely to have contributed to the CSE-induced effects presented in this study, since LPS concentrations in the CSE were hardly detectable and far below the concentrations needed to induce ASM cell proliferation (not shown). This is in accordance with previous studies demonstrating that the LPS concentration in CSE is very low and that neutralisation of LPS in CSE, using polymyxin B, does not affect the CSE-induced IL-8 release by human macrophages [55]. In addition, we investigated the effect of combined CSE and LPS treatment on ASM cell proliferation, since both factors may be involved simultaneously in exacerbations of COPD. However, no additive effects were observed, clearly indicating that both stimuli act via common pathways, as previously also suggested by others [55].

ASM cells display phenotypic plasticity, characterized by reversible changes in contractile, proliferative and synthetic characteristics, and governed by a variety of growth factors, cytokines, G-protein-coupled receptor agonists and ECM proteins [33-35,38,56-59]. In vitro, smooth muscle-specific contractile protein expression is reduced in response to serum-rich media or growth factors, leading to a decrease in contractility, whereas the proliferative capacity is increased [33,35,57,58]. Previous studies have shown that ERK 1/2 and p38 MAP kinase are importantly involved in PDGF-induced proliferation and hypocontractility of ASM [33,35]. Indeed, activation of ERK 1/2 has been shown to increase the expression of cyclin D1, a key regulator of G_1 _phase cell cycle progression [60,61] and to play a fundamental role in ASM cell proliferation [60,62-64]. p38 MAP kinase activation has also been shown to contribute to ASM cell cycle progression and proliferation [33,35,65-68], although this may depend on the mitogen used [65,68]. The present study demonstrated that both CSE and LPS induce phosphorylation of ERK 1/2 and p38 MAP kinase as well as increased expression of cyclin D1 in BTSM cells, whereas inhibition of ERK 1/2 and p38 MAP kinase prevented the CSE- and LPS-induced proliferation of these cells. As a possible mechanism that may be involved, CSE was recently shown to induce ERK 1/2 and p38 MAP kinase phosphorylation through NADPH oxidase-induced reactive oxygen species (ROS) formation in human ASM cells [69]. NADPH oxidase has previously also been shown to be involved in proliferative effects of TGF-β_1 _in these cells [70].

Expression of TLR4 receptors [31,32] and LPS-induced ERK 1/2 and p38 MAP kinase phosphorylation [31,71] in ASM cells have previously been reported as well. Remarkably, in rabbit ASM, it was shown that LPS-induced ERK 1/2 and p38 MAP kinase activation had opposing effects on LPS-induced hypercontractility [31]. The LPS-induced hypercontractility of rabbit ASM preparations seems to be at variance with our observation of an LPS-induced hypocontractility of BTSM. Difference in duration of LPS treatment (24 h vs 8 days in our study) as well as species differences could possibly underlie this difference. Indeed, a previous study from our lab indicated that at least 4 days of treatment with FBS was required to induce a proliferative BTSM phenotype with a significant decrease in contractility [33]. A hypocontractile ASM phenotype has also been observed after long-term incubation of ASM preparations with other growth factors, including PDGF and IGF-1 [33] as well as with pro-proliferative ECM proteins, such as collagen I and fibronectin [34]. It has been demonstrated that the reduced contractility induced by growth factors and ECM proteins is accompanied by reduced expression of contractile proteins, such as sm-myosin, calponin and sm-α-actin [34]. Such a mechanism could also underlie CSE- and LPS-induced hypocontractility of BTSM. Thus, CSE as well as LPS reduced the maximal contractile response to both a receptor-dependent (methacholine) and a receptor-independent (KCl) stimulus, indicating that post-receptor alterations such as reduced contractile protein expression are likely to be involved.

## Conclusions

In conclusion, our in vitro data provide evidence that both CSE and LPS may contribute to airway remodelling in COPD through direct effects on ASM cells causing a proliferative phenotype that may be involved in increased ASM mass in this disease.

## Competing interests

The authors declare that they have no competing interests.

## Authors' contributions

TP carried out the proliferation and the contractility studies, performed the statistical analysis, participated in conceiving and designing the study and drafted the manuscript. RG participated in designing the study and revised the manuscript. AHL performed western analysis and participated in the proliferation studies. RS performed western analysis. MvdT participated in designing the study. JZ participated in conceiving and designing the study and revising the manuscript. HM conceived the study, participated in designing the study and revised the manuscript. All authors have read and approved the final manuscript.
